# Comparative genomic analysis of a naturally born serpentized pig reveals putative mutations related to limb and bone development

**DOI:** 10.1186/s12864-021-07925-3

**Published:** 2021-08-28

**Authors:** Yankai Jiang, Xinyue Cao, Haibin Wang

**Affiliations:** 1 Department of Joint Surgery, The Second Hospital of Shandong University, Jinan, 250033 Shandong China; 2 School of Medicine and Nursing, Dezhou University, Dezhou, 253023 Shandong China

**Keywords:** Serpentized pig, Hindlimb, Bone, Genome sequencing, Mutations

## Abstract

**Background:**

It is believed that natural selection acts on the phenotypical changes caused by mutations. Phenotypically, from fishes to amphibians to reptiles, the emergence of limbs greatly facilitates the landing of ancient vertebrates, but the causal mutations and evolutionary trajectory of this process remain unclear.

**Results:**

We serendipitously obtained a pig of limbless phenotype. Mutations specific to this handicapped pig were identified using genome re-sequencing and comparative genomic analysis. We narrowed down the causal mutations to particular chromosomes and even several candidate genes and sites, such like a mutation-containing codon in gene *BMP7* (bone morphogenetic protein) which was conserved in mammals but variable in lower vertebrates.

**Conclusions:**

We parsed the limbless-related mutations in the light of evolution. The limbless pig shows phenocopy of the clades before legs were evolved. Our findings might help deduce the emergence of limbs during vertebrate evolution and should be appealing to the broad community of human genetics and evolutionary biology.

**Supplementary Information:**

The online version contains supplementary material available at 10.1186/s12864-021-07925-3.

## Background

Both mutations and environmental factors shape the phenotype of living organisms. When the environmental effects are controlled, mutations become the most plausible trigger of phenotypical changes. Indeed, during the long-term evolution, mutations are the source of natural selection [1, 2]. Based on the neutral theory of evolution [3, 4], most novel mutations are deleterious and already eliminated by purifying selection. The more essential a gene is, the stronger the purifying selection would be. As a consequence, in the inter-species comparison of orthologous genes, the essential genes tend to show less divergence (higher sequence similarity) than the unimportant genes [5, 6]. From another aspect, a novel mutation on an essential gene, if deleterious, would severely reduce the fitness and even cause the death of an individual, which maintains the high conservation level of sequence among populations.

Given the strong connection between the cause (mutation), effect (fitness change), and consequence (individual eliminated), one could infer the effect of a mutation from the mutation spectrum within a species or a population. A population consists of numerous individuals. When a novel mutation (in an individual) is beneficial, the carrier would have higher chance of survival or reproduction and therefore this mutation would increase its allele frequency (AF) during evolution [7, 8]. If a deleterious mutation occurs, the carrier is less viable so that the AF of this mutation would be constantly low or sometimes this mutation would be directly eliminated from the population. At species level (which is beyond population), beneficial mutations might be eventually fixed in all individuals of a species, leading to the fixation of divergent sites between two species [9, 10]. The deleterious and beneficial mutations show completely different evolutionary traces. Therefore, analyzing the mutations in the light of evolution is helpful in distinguishing which sets of mutations are beneficial or deleterious, and might facilitate the identification of causal mutations (for a phenotype) from the sea of randomly occurred mutations.

Undoubtedly, the emergence of limbs is a milestone during the evolution of vertebrates. From the very basal water-living fishes to the most recently diverged mammalian branch (also the most successful branch), landing should be the most significant event among all the evolutionary steps, which brings about numerous advantages. Thus, understanding the evolutionary traces and molecular mechanisms of limb development is of grave importance. Experimental biologists indeed find a series of candidate genes (proteins) responsible for that. To date, the broadly conserved regulators related to limb or bone development include the T-box family transcription factor (TBX4) [11, 12], the bone morphogenetic proteins (BMP) [13, 14] (especially two members BMP7 [15] and BMP2 [16]), downstream effector SMAD [17, 18], PTX1 [19], R-spondin secreted ligand RSPO [20], forehead transcription factor FOXF [21], and HOX [22].

The above discoveries are either found in humans or some well-established model organisms. However, one still wonders if the defects in limbs could take place in natural environment or in a less-studied organism. Although genetic manipulation is not feasible in non-model organisms, the evolutionary and comparative genomic analyses across multiple species still make sense in the absence of functional verification [23]. In this study, we report a naturally born farmed pig (*Sus scrofa*) without hindlimbs, similar to the serpentized phenotype [24]. Although finding deformities in farmed pigs are not rare, few cases have been thoroughly investigated by bioinformatic analysis. By considering the Mendelian independent assortment law, we also narrowed down the specific mutations to particular chromosomes and even several candidate genes and sites, such like a mutation-containing codon in gene *BMP7* which was conserved in mammals but variable in birds, reptiles, amphibians, and fishes. Our study shows that the limbless phenotype is not confined to a few model organisms but could be more general across mammals, we also parse the specific mutations in the light of evolution. We propose the functional role of many candidate genes and sites in hindlimb development. Our work should be valuable to the human genetics as well as evolutionary biology community.

## Results

### General description of a naturally born limbless pig

**Fig. 1:**
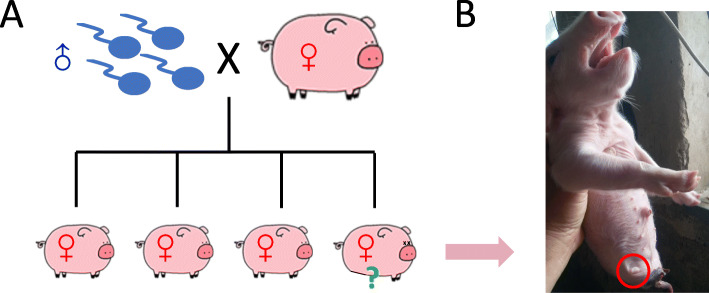
General description of the handicapped pig. (**A**) A diagram of the pedigree. The female F0 was artificially fertilized. Four female piglets (F1) were born. One F1 individual did not have hindlimbs. This handicapped pig was denoted as H. (**B**) A photo of the newly born handicapped pig (1 day, June 30th, 2020). Red circle emphasized the position of hindlimb. The clipart images are made by ourselves

We obtained a naturally born handicapped pig from a farm near Yantai city, China (see Methods for details). This handicapped pig without hindlimbs is one of the four female piglets (F1) delivered by a female pig (F0) who has previously been artificially fertilized (Fig. 1A and B). The morphology of this handicapped pig resembles the well-known serpentized phenotype (Fig. 1B). Further X-ray photos confirmed that the leg bones are undeveloped (Additional file 1: Fig. S1).

The handicapped pig lived for 30 days and died of natural causes or causes due to its deformities. The pig’s hind leg deformities made it harder to function like normal pigs, including walking and eating (Additional file 2: supplementary data). The failure of the hindlimb development was our main focus in this study and not the cause of the early death to investigate the if there was a link between phenotype and genotype.


**Additional file 2.** Video showing the clumsiness of the handicapped pig.


### Genomic sequencing reveals numerous mutations

**Fig. 2:**
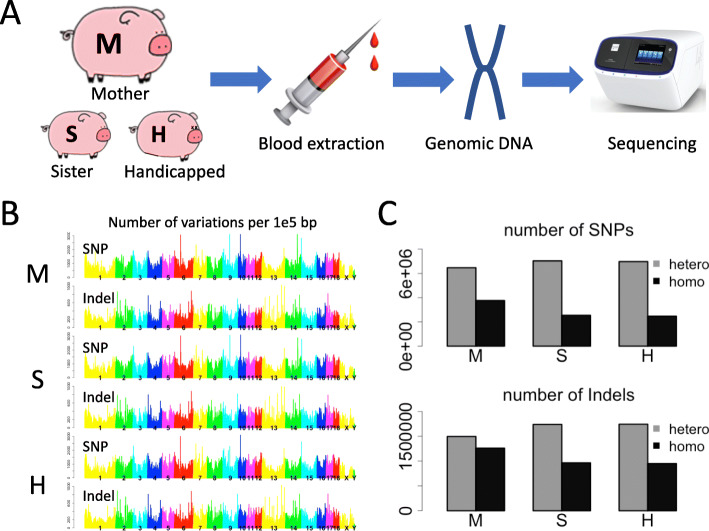
Procedure of genome sequencing and the profile of variations. (**A**) Blood was extracted from the tails of the mother (M), sister (S), and the handicapped (H) pig. Genomic DNA was extracted and next generation sequencing was performed. (**B**) Density (variations per 100,000 bps) across the pig genome. Each chromosome was shown in a distinct color. (**C**) Comparison of heterozygous and homozygous variants: heterozygous and homozygous variations for detected SNPs and Indels. M, mother; S, sister; H, handicapped pig. The clipart images are made by ourselves

Intuitively, the phenotype should be governed by the genotype as well as the external factor (environment). Indeed, there are a wide range of teratogenic agents that can affect limb morphogenesis and cause vascular disruption on limbs. However, since the pigs were subjected to the same environmental conditions, it is hard for us to tell how this particular pig could be the only handicapped one among all the offspring. Therefore, we could only try to analyze the contribution of genotype to the phenotype. We sequenced the genomic DNA of the female F0 individual (denoted as M, standing for mother), the handicapped individual (denoted as H), and another F1 individual (denoted as S, standing for sister) (Fig. 2A). After mapping the DNA-sequencing reads to the reference genome, numerous bona fide mutations are found (see Methods). Briefly, M has 13,212,738 variation sites, S has 12,503,665, and H has 12,355,632. The variation types include both SNPs (point mutations) and Indels (potential frame-shift variations), covering all the chromosomes of the pig genome (Fig. 2B). Notably, when talking about variations in diploid species, it should be clarified whether a variation is homozygous or heterozygous. For deleterious mutations, homozygosity is possibly lethal, so the commonly observed deleterious mutations are heterozygous. One could conceive that Indels are more harmful that point mutations (SNPs), and accordingly, in our data, Indels are more likely to be heterozygous than SNPs (Fig. 2C). This result is expected and conform with known theories.

### Profiling the variations specific to the handicapped pig

**Fig. 3:**
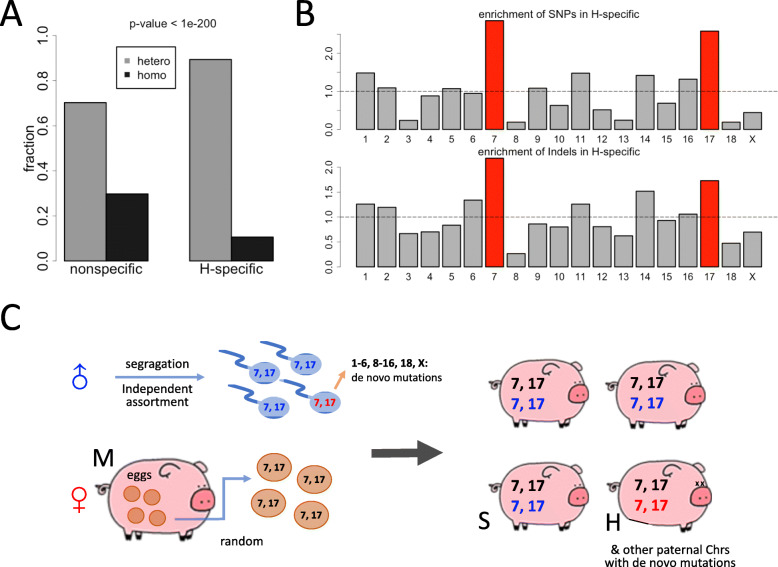
Inference of the independent assortment process during meiosis. (**A**) “H” denotes the handicapped pig. This barplot showed the fractions of heterozygous/homozygous variations for the H-specific variations and H-nonspecific variations. “H-specific” means appearing in H but not appearing in either M or S. *P*-value was calculated from Chi-square test. (**B**) Enrichment of variations in the H-specific group on each chromosome. SNPs and Indels were shown separately. Chromosomes 7 and 17 had the highest enrichment of H-specific variations. As explained in Methods, here the Y-axis is the enrichment = F_Hi_/F_Ni_, 1 ≤ i ≤ 18 or i = chrX. The horizontal line is Y = 1, which indicates equal frequency among H (H-specific mutations) and N (non-specific mutations) (C) The putative independent assortment process inferred from the enrichment of H-specific variations. For sperms, the handicapped pig inherited a specific set of chromosomes 7 and 17 (colored in red), while the normal offspring inherited another set of chromosomes 7 and 17 (colored in blue). For eggs, the combination was random. Apart from the specific mutations inherited from chromosomes 7 and 17, the handicapped pig might also have de novo mutations. The clipart images are made by ourselves

To find putative variations accountable for the handicapped phenotype, we should not focus on the variant sites shared by M, S, and H. Those sites are fixed in this pedigree and should not cause handicap in only one individual. Instead, the variant sites specific to the handicapped pig are more likely to explain this unique phenotype. We found that 8.7% (1,079,634 out of 12,355,632) variations in H belong to H-specific category (Additional file 3: Table S1) and the remaining 91.3% is shared with at least one of other two sequenced individuals. Interestingly, H-specific variations had a significantly higher fraction of heterozygous sites (89.4%) compared to non-specific variations (70.2%) (Fig. 3A). Again, this is plausible as the non-specific variations are shared in this pedigree so that they might already be fixed during evolution.

According to the Mendelian segregation and independent assortment laws, each F1 individual has a 0.5 chance to inherit a particular paternal chromosome. In our case, the H-specific variations could only be acquired from the independent assortment of paternal alleles or de novo germline mutations. The number of de novo mutations (under a rate of ~ 10^− 8^ per nucleotide per generation, in mammals) [1] was negligible compared to the specific mutations contained in a particular paternal allele. For example, the de novo mutations carried by each individual is usually less than 100 (under a rate of ~ 10^− 8^), while the paternal allele versus maternal allele could have millions of nucleotides to be different, just like the millions of different sites between two randomly picked human individuals. Therefore, the density profile of H-specific variations could tell us which paternal chromosomes were uniquely transmitted to this handicapped individual. We calculated the frequency of variations appearing in each chromosome, and compared the frequencies of H-specific and non-specific sites (see Methods). We found that the H-specific variations are significantly enriched in chromosome 7 and chromosome 17 (Fig. 3B). This is a strong indication that individual H inherited a set of chromosomes 7 and 17 from paternal alleles while the other sister analyzed inherited another set of chromosomes 7 and 17 from the father (Fig. 3C). Indeed, de novo mutations were also contained in the variation profile, however, as we have stated, the number of de novo mutations was too small to affect the global distribution of variations. Therefore, there is high confidence to claim that the chromosomes 7 and 17 of this handicapped pig were “differentially” inherited from the paternal side due to independent assortment.

### Evolutionary analysis on the H-specific variations

**Fig. 4:**
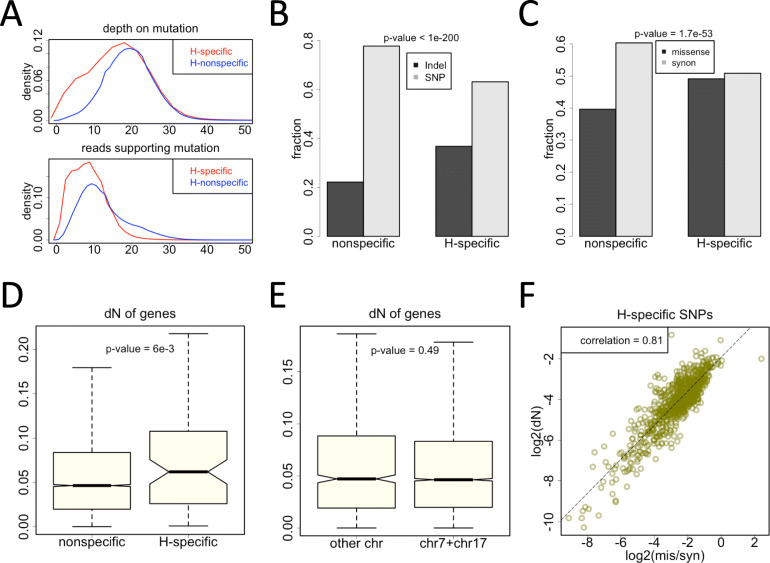
Features of H-specific variations. (**A**) Density plot of sequencing coverage on mutation sites. (**B**) Fractions of Indels and SNPs in H-specific and nonspecific sites. P-value was calculated by Chi-square test. (**C**) Fractions of missense and synonymous SNPs in H-specific and nonspecific sites. P-value was calculated by Chi-square test. (**D**) For the H-specific or nonspecific sites, their unique genes were studied separately. dN (missense substitution rate) values were displayed by boxplot. P-value was calculated by KS test. (**E**) Comparison of dN values of genes on chromosomes 7 and 17 versus other chromosomes. P-value was calculated by KS test. (**F**) Spearman’s correlation (*p*-value <2E-16) between the dN values of genes and the ratio of missense to synonymous SNPs. Only the H-specific SNPs were used to calculate the missense to synonymous ratio on each gene

To have a better knowledge of the functional impact of the H-specific variations, we intend to analyze these mutations in the light of evolution [23]. First, we show that these mutation sites are covered by adequate reads and that the numbers of reads supporting the variation (alternative allele) are sufficiently high (Fig. 4A). This ensures that the variations especially the H-specific ones were not produced from artefacts. The mean depth of mutation sites is ~ 20 and the mean reads supporting the mutated allele is ~ 10. Given a sequencing error rate of 1%, the observed mutation is not likely to be the artefact of sequencing error (binomial test *p*-value <1E-20 under depth = 20 and mutated allele = 10). Therefore, the observed variants should be genuine mutation sites. Next, we found that the H-specific variations have higher fractions of Indels compared to non-specific variations (Fig. 4B). Indels are generally more deleterious than SNPs, and therefore the Indels specific to H might play a role in shaping the handicapped phenotype. Similarly, among the SNPs in coding regions, the H-specific SNPs have higher fraction of missense mutations compared to non-specific SNPs (Fig. 4C). In coding regions, missense mutations have stronger functional consequence than synonymous mutations due to the change in amino acids. Again, the higher fraction of missense SNPs for the H-specific mutations indicates that these impactful variations might contribute to the handicapped phenotype.

We next focus on the conservation level of each gene. The dN (nonsynonymous substitution rate) values of genes are used to measure the divergence between the pig and ox orthologous genes (see Methods). Lower dN value represents higher conservation level for a gene. If a gene only has H-specific variations but not the non-specific variations, then this gene is defined as H-specific gene (Additional file 4: Table S2), and the other genes with variations are defined as non-specific genes. We found that H-specific genes are significantly less conserved than the non-specific genes (Fig. 4D). This difference in conservation level is not caused by the genes on chromosomes 7 and 17 (Fig. 4E). Intuitively, the genes with the highest conservation level are usually those constitutionally expressed genes with great importance, and mutations on these most essential genes might be lethal and eliminated by natural selection. Now that we observed the H-specific variations in this viable (but with lower fitness) individual, suggesting that these H-specific variations must not be so deleterious to cause a lethal phenotype. This assumption well confirms our observation that the H-specific genes are less conserved (Fig. 4D).

Nevertheless, we still found an interesting pattern for the H-specific SNPs. We focused on the genes with both missense and synonymous SNPs (H-specific ones), and calculated the missense to synonymous ratio (denoted as “mis/syn”) for each gene. Strikingly, the mis/syn ratio of genes (the number of missense mutations in a gene divided by the number of synonymous mutations in this gene) is significantly correlated with the conservation level of genes (Fig. 4F). This is to say, conserved genes tend to have fewer H-specific missense mutations (compared to the synonymous mutations, which are regarded as neutral). As we mentioned above, conserved genes are susceptible to the deleterious missense mutations, so these missense mutations are suppressed in these genes. From another aspect, these observed H-specific missense mutations should be paid special attention to. These H-specific mutations are non-lethal so that they escaped the strongest purifying selection and are maintained to the present, but they might be associated with the limbless phenotype (which is of lower fitness) observed in the handicapped pig.

### Putative candidate genes related to limb development

The H-specific variations are composed of two parts. Most of the H-specific variations are supposed to be inherited from particular paternal alleles, and only a minor fraction of H-specific variations belongs to de novo mutations that are randomly dispersed across the genome. Here we would mainly discuss the candidate genes (the genes previously described to be involved in limb development) which are located on chromosomes 7 and 17. These genes contribute most to the H-specific variations. Some variations that could not be explained by independent assortment are attributed to the possible de novo mutations.

**Fig. 5:**
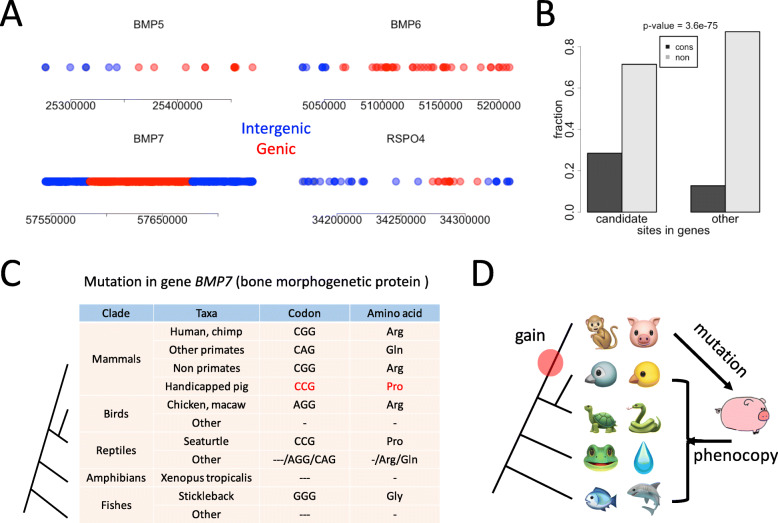
Candidate genes relevant to hindlimb development on chromosomes 7 and 17. (**A**) The distribution of H-specific variations on or near the candidate genes. Red dots represented variations in gene regions and blue dots represented variations in intergenic regions. (**B**) The fractions of conserved nucleotides of the variation sites on the candidate or non-candidate genes. If the reference sequence of pig was identical to the orthologous nucleotide in human, then this position is denoted as conserved. (**C**) An H-specific missense mutation in gene *BMP7* (bone morphogenetic protein), leading to an arginine (CGG) to proline (CCG) change in handicapped pig. This position was relatively conserved in mammals but was variable in birds, reptiles, amphibians, and fishes. (**D**) Putative evolutionary trajectory of the candidate gene controlling hindlimb development. This trait might be obtained in the common ancestor of mammals. A mutation in this gene lead to the phenotype of the handicapped pig. The clipart images are made by ourselves

Among the various hindlimb-related genes we introduced, *BMP5*, *BMP6* and *FOXF2* are located in chromosome 7, *BMP2*, *BMP7* and *RSPO4* are located in chromosome 17. Note that the most “promising” gene *TBX4* is located in chromosome 12, and very few H-specific variations are found in *TBX4*. Thus, we regard the six genes (*BMP2*, *BMP5*, *BMP6*, *BMP7*, *FOXF2*, and *RSPO4*) as candidate genes. We show that many of the variations related to candidate genes are intergenic (Fig. 5A), and for the genic variations, most of them are intronic or located in UTRs. Our assumption is, if these six candidate genes really play essential roles in driving the limbless phenotype, then they might exhibit different mutation profiles (differences in the fractions of conserved sites) when compared to other non-candidate genes. This is intuitive because the essential residues are usually conserved and constrained (maintained) by purifying selection. If the essential sites are mutated, the consequence might be more severe than the mutations on non-essential sites. Since we want to attribute the handicapped phenotype to the particular genotype, we should find any possible patterns on these H-specific sites and candidate genes.

We first compared the six candidate genes with other genes on chromosome 7 and 17. The mutation sites could be classified into two categories regarding whether the genomic nucleotide is conserved between pig and human, that is, the conserved group and non-conserved group. By this way, we looked at the fractions of conserved nucleotides. We found that variations in candidate genes have a higher fraction of conserved nucleotides than the variations in non-candidate genes (Fig. 5B). This result is meaningful because the mutations on more conserved sites are more likely to cause deleterious effects.

However, although the mutations on candidate genes exhibit higher conservation level, we only found missense mutations in gene *BMP7*. There are two H-specific missense mutations in gene *BMP7*. One caused an arginine to proline change at protein position 73 (Arg73Pro, chr17:57676401, 18 reads covered, 10 of which supported the mutated allele), another caused an alanine to threonine change at protein position 148 (Ala148Thr, chr17:57676177, 28 reads covered, 11 of which supported the mutated allele). From the reads covering the sites, it is easy to tell that these H-specific SNPs are heterozygous: given a sequencing error rate of 1%, the observed mutation is not likely to be the artefact of sequencing error (binomial test *p*-value <1E-20 under depth = 20 and mutated allele = 10). Therefore, it should be a reliable heterogenic SNP. We then looked at the genome alignment in vertebrate species, and found an intriguing pattern on site Arg73Pro (CGG to CCG) (Fig. 5C). In most mammals including human, chimpanzee, and non-primates (including pig), the reference genome is CGG that encodes Arg. This site is mutated to CCG and encodes Pro in the handicapped pig. In contrast, this site is highly variable in birds, reptiles, amphibians, and fishes (Fig. 5C). This site could be summarized/described as “conserved in mammals but not conserved in lower vertebrates”. Specially, seaturtle has the same sequence as the handicapped pig, which is a CCG codon encoding Pro. It is plausible that the development of normal mammalian hindlimbs was gained in the common ancestor of mammals but reversed in the handicapped pig, leading to the phenotype mimicking some of the lower vertebrates (Fig. 5D).

## Discussion

During long-term evolution, highly deleterious mutations have been eliminated and could no longer be seen at present time [25]. Beneficial mutations increase their frequency in natural populations [26] but the net gain of fitness is so subtle to be observed at phenotypical level [27]. This dilemma adds difficulty to the functional verification of mutations and makes the evolutionary theories hard to guide experimental biologists [28]. Our work proposes putative solution to this dilemma by obtaining the genetic information from (otherwise) extinct nodes in the tree of life, providing precious information (or direct observation) of the fitness cost of the deleterious mutations.

In most phylogenetic trees, the topology is determined by the sequences of the extant species at the end of each branch. The ancestral nodes have already gone extinct so that the information is missing. However, the genome sequences of extinct species, if available, will be extremely helpful in the inference of adaptation and function, and this information could even calibrate the phylogeny. For instance, the sequences of extinct Neanderthals (*Homo neanderthalensis*) and Denisova help researchers define the mutations related to cognition that might cause the extinction of the ancient human species [29]. Intriguingly, the extinction process not only took place in ancient times, it takes place all the time.

One could regard this handicapped pig as an extinct individual and the living pigs as extant individuals. Our study is to perform comparative genomics between the sequences of an extinct individual and extant individuals, which is similar to the comparison between *H. sapiens* and *Homo neanderthalensis* or *Homo erectus*. We found H-specific missense mutations that might be accountable for the disadvantageous phenotype. Here, we should admit that we have presumed that the synonymous mutations are neutral, as many studies did [30, 31]. However, this might not always be the case since the synonymous codon usage is also subjected to weak selection as numerous papers have already demonstrated in all kingdoms of organisms [32–43].

Another fortune of this study is that we found particular candidate genes and sites that potentially related to the limbless phenotype, such as the Arg73Pro site on gene *BMP7*. Among the numerous H-specific mutations, Arg73Pro site is one of limited mutations in coding region and caused amino acid change. More importantly, the sequence on this site exhibits clear clade-specific pattern during vertebrate evolution (relatively conserved in mammals but variable in lower vertebrates). This made it the best candidate to account for the limbless phenotype. We also found other candidate mutations (not missense mutations) on the same chromosome (chr17) like those in genes *BMP2* and *RSPO4*. These mutations are provided together with the H-specific variations in Additional file 3: Table S1. These mutations, although not missense mutations, could also be relevant. But the detailed mechanism is uninvestigated at this stage, possibly interacting with other regulators and epigenetic or post-transcriptional pathways [44–48].

Interestingly, while genes *BMP2*, *BMP7*, and *RSPO4* are located in chromosome 17 in pig, these three genes are also located in the same chromosome (chr20) in human. That means that the linkage of these three genes is highly conserved during evolution. Since genes linked within the same chromosome are more likely to have interaction and co-regulation, the linkage of *BMP2*, *BMP7*, and *RSPO4* in mammals might be associated with the epistasis and regulatory network among these genes. During evolution, the disruption of this linkage might break the regulatory chain in bone and limb development and therefore is purged by purifying selection.

**Fig. 6:**
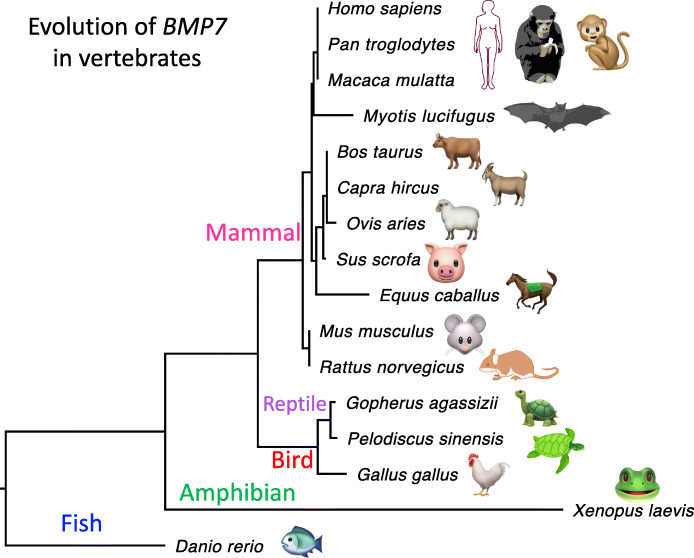
The phylogenetic tree of *BMP7* in vertebrates. The tree is based on protein sequences with maximum likelihood. The branch length is scaled to evolutionary distance. The clipart images are made by ourselves

In addition, there is a minor but interesting point that might explain the importance of *BMP7* in mammals. The phylogenetic tree of gene *BMP7* in vertebrates shows an extremely low evolution rate in mammals compared with other vertebrate clades like fish, amphibian, reptile, and bird (Fig. 6). This indicates that *BMP7* is highly conserved in mammals but less conserved in lower vertebrates. The selection constraint on mammalian *BMP7* makes the missense mutations in *BMP7* deleterious.

We admit that the deformed phenotype is not necessarily caused by mutations. On one hand, we are fully aware that the phenotype is caused by the combinatory effect of genotype and environment. On the other hand, we do not wish to overstate the rarity of this handicapped pig as we know that these kinds of animals are frequently seen in worldwide farms and that pig is already used as a model animal. However, in our study, since all pigs are subjected to the same environmental conditions, it is hard for us to tell how this particular pig could be the only handicapped one among all the piglets. Therefore, we could only surmise this handicapped pig might have inherited specific alleles from her parents (due to independent assortment) or have suffered from de novo germline mutations. This is the most intuitive and plausible explanation. In addition, we found relevant mutations with evolutionary significance by whole genome sequencing. Our efforts should of great biological significance.

## Conclusion

We parsed the limbless-related mutations in the light of evolution. The limbless pig shows phenocopy of the clades before legs were evolved. Our findings might help deduce the emergence of limbs during vertebrate evolution and should be appealing to the broad community of human genetics and evolutionary biology.

## Methods

### Sample collection

The pigs raised in a farm in Yantai are subjected to intensive farming. The feeding trough is filled with feed all the time. Feed would be automatically added to the trough when it goes empty. The new-born pigs are vaccinated with foot-and-mouth disease (FMD) vaccine. Usually, one female pig would deliver five to ten offspring one time. The female F0 pig was artificially fertilized. Four female piglets were born on 30th June, one of which did not have hindlimbs. We did not perform a necropsy. As we have surmised, this pig has deficiency in movement and has disadvantage in competing for food. The lower fitness might be the cause of early death. The other pigs were living well.

DNA was extracted from the tail blood. Next generation sequencing was accomplished by “Geneseeq” in Nanjing, China. The platform was Illumina Hiseq 2500, 150 bp, pair-ended.

### Reference genome

The direct links of the database are given as follows.

Pig (*Sus scrofa*) genome sequence: ftp://ftp.ensembl.org/pub/release-101/fasta/sus_scrofa/dna/Sus_scrofa.Sscrofa11.1.dna_sm.toplevel.fa.

Pig (*Sus scrofa*) genome annotation file: ftp://ftp.ensembl.org/pub/release-101/gtf/sus_scrofa/Sus_scrofa.Sscrofa11.1.101.gtf.gz.

Ox (*B. taurus*) genome sequence: ftp://ftp.ensembl.org/pub/release-101/fasta/bos_taurus/dna/Bos_taurus.ARS-UCD1.2.dna_sm.toplevel.fa.gz.

Ox (*B. taurus*) genome annotation file: ftp://ftp.ensembl.org/pub/release-101/gtf/bos_taurus/Bos_taurus.ARS-UCD1.2.101.gtf.gz.

Human (*H. sapiens*) genome sequence: ftp://ftp.ensembl.org/pub/release-101/fasta/homo_sapiens/dna/Homo_sapiens.GRCh38.dna_sm.toplevel.fa.gz.

Human (*H. sapiens*) genome annotation file: ftp://ftp.ensembl.org/pub/release-101/gtf/homo_sapiens/Homo_sapiens.GRCh38.101.gtf.gz.

### Mapping and variant calling

Reads mapping was accomplished by software BWA mem [49] (version 0.7.17-r1188). Unique mappers were maintained. Variant calling was done by GATK [50] (version 3.7). Reads depth at a given position was called by Samtools [51] (version 1.12). Given a variant in the genome, software SnpEff [52] (version 4.2) was used to annotate the effect of the variant, including whether it is a SNP or Indel, whether it is located in gene region, whether it hit coding region, or whether it changes amino acid. The information of heterozygosity was provided in the variant calling result. For bi-allelic sites, “0/1” represents heterozygous variants and “1/1” represents homozygous sites. The reads count of two alleles could also be extracted from the output file of variant calling. We further require a mutation site to have reads coverage > 10 and alternative reads count > 3. Under these criteria, the variations obtained were not likely to be artefacts like sequencing errors (*p*-value <1e-10).

### Conservation and divergence

A tool named liftOver [53] was able to convert the orthologous sites across species. For example, if species-A is the anchor species of interest (pig), by converting the variant coordinate in species-A to the genomic coordinate in species-B, one could know the orthologous nucleotide in species-B by using Bedtools [54]. The conservation pattern between species-A and species-B could be told from the nucleotides on orthologous sites.

Next, the divergence between two species is measured by dN and dS, named missense and synonymous substitution rates. Each pair of orthologous gene (between two species) has one set of dN and dS values. The exact values were calculated by PAML [5]. Usually, conserved and essential genes have lower dN and dS values.

The phylogenetic tree of BMP7 is based on protein sequences from UniProt website [55]. Sequences are aligned [56–58] and constructed with maximum likelihood [59].

### Enrichment of H-specific mutations

Let “H” denote H-specific mutations and “N” denote non-specific mutations.

Let “F” represent the frequency, which is calculated as Fxi = xi/(× 1 + × 2 + .. +xn), where 1 ≤ i ≤ n.

For simplicity, here we presume there are 18 chromosomes numbered 1 ~ 18:

For H-specific mutations, F_Hi_ = H_i_/(H_1_ + ... + H_18_), 1 ≤ i ≤ 18.

For non-specific mutations, F_Ni_ = N_i_/(N_1_ + ... + N_18_), 1 ≤ i ≤ 18.

The enrichment of chromosome i = F_Hi_/F_Ni_, 1 ≤ i ≤ 18.

The significance is calculated by Chi-square test:

Let “H” denote H-specific mutations and “N” denote non-specific mutations. For example, to test the significance of chromosome 7, we need four numbers: H_7_, N_7_, H_[other_chr]_, and N_[other_chr]_. The p-value was calculated by Chi-square test using these four numbers. *P*-value < 0.05 was regarded as significant.

### Data deposition

The DNA-sequencing data were deposited to the Genome Sequence Archive (GSA) under accession number PRJCA003678.

## Supplementary Information

**Additional file 1: Fig. S1.** X-ray (side view and vertical view) of the handicapped pig (14 days, July 13th, 2020). Human hands were inevitably included in this graph.

**Additional file 3: Table S1.** List of H-specific variations.

**Additional file 4: Table S2.** List of H-specific genes.

## Data Availability

Pig (*Sus scrofa*) genome sequence: ftp://ftp.ensembl.org/pub/release-101/fasta/sus_scrofa/dna/Sus_scrofa.Sscrofa11.1.dna_sm.toplevel.fa. Pig (*Sus scrofa*) genome annotation file: ftp://ftp.ensembl.org/pub/release-101/gtf/sus_scrofa/Sus_scrofa.Sscrofa11.1.101.gtf.gz. Ox (*B. taurus*) genome sequence: ftp://ftp.ensembl.org/pub/release-101/fasta/bos_taurus/dna/Bos_taurus.ARS-UCD1.2.dna_sm.toplevel.fa.gz. Ox (*B. taurus*) genome annotation file: ftp://ftp.ensembl.org/pub/release-101/gtf/bos_taurus/Bos_taurus.ARS-UCD1.2.101.gtf.gz. Human (*H. sapiens*) genome sequence: ftp://ftp.ensembl.org/pub/release-101/fasta/homo_sapiens/dna/Homo_sapiens.GRCh38.dna_sm.toplevel.fa.gz. Human (*H. sapiens*) genome annotation file: ftp://ftp.ensembl.org/pub/release-101/gtf/homo_sapiens/Homo_sapiens.GRCh38.101.gtf.gz. Genome transfer tools: http://hgdownload.soe.ucsc.edu/goldenPath/susScr11/liftOver/. The DNA-sequencing data were deposited to Genome Sequence Archive (GSA) under accession number PRJCA003678.

## Abbreviations

*BMP*: bone morphogenetic protein
*PTX1*: Paired Like Homeodomain 1
*RSPO*: R-Spondin
*FOXF*: Forkhead Box F
*HOX*: Homeobox
AF: allele frequency
SNP: single nucleotide polymorphism
dN: nonsynonymous substitution rate
M: mother
S: sister
H: handicapped pig
