# Supplementary figures and images for: Comparative genomic analysis of a naturally born serpentized pig reveals putative mutations related to limb and bone development
Source: BMC Genomics. 2021 Aug 28;22:629. doi: 10.1186/s12864-021-07925-3 (PMC8399796; doi:10.1186/s12864-021-07925-3)

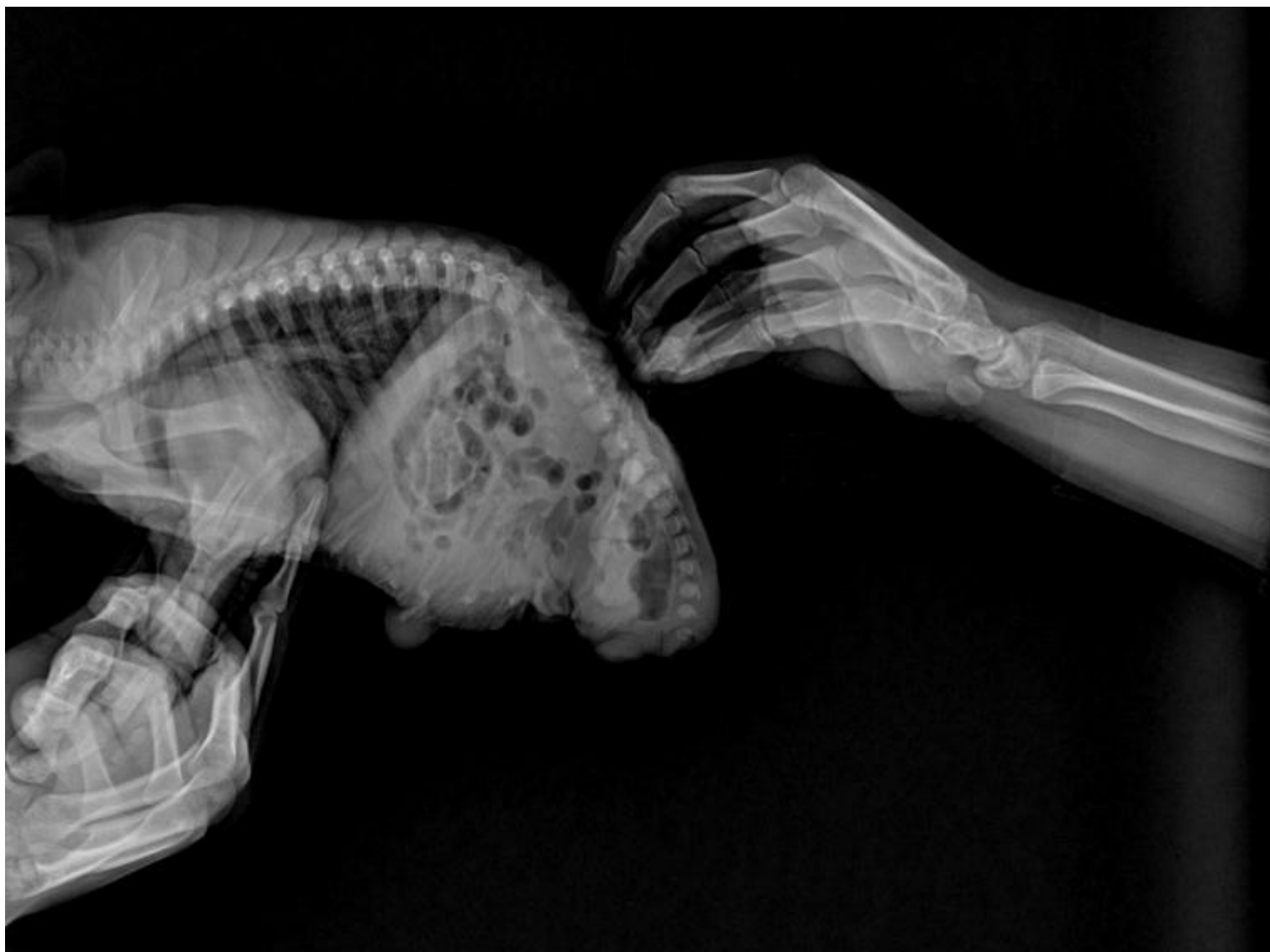

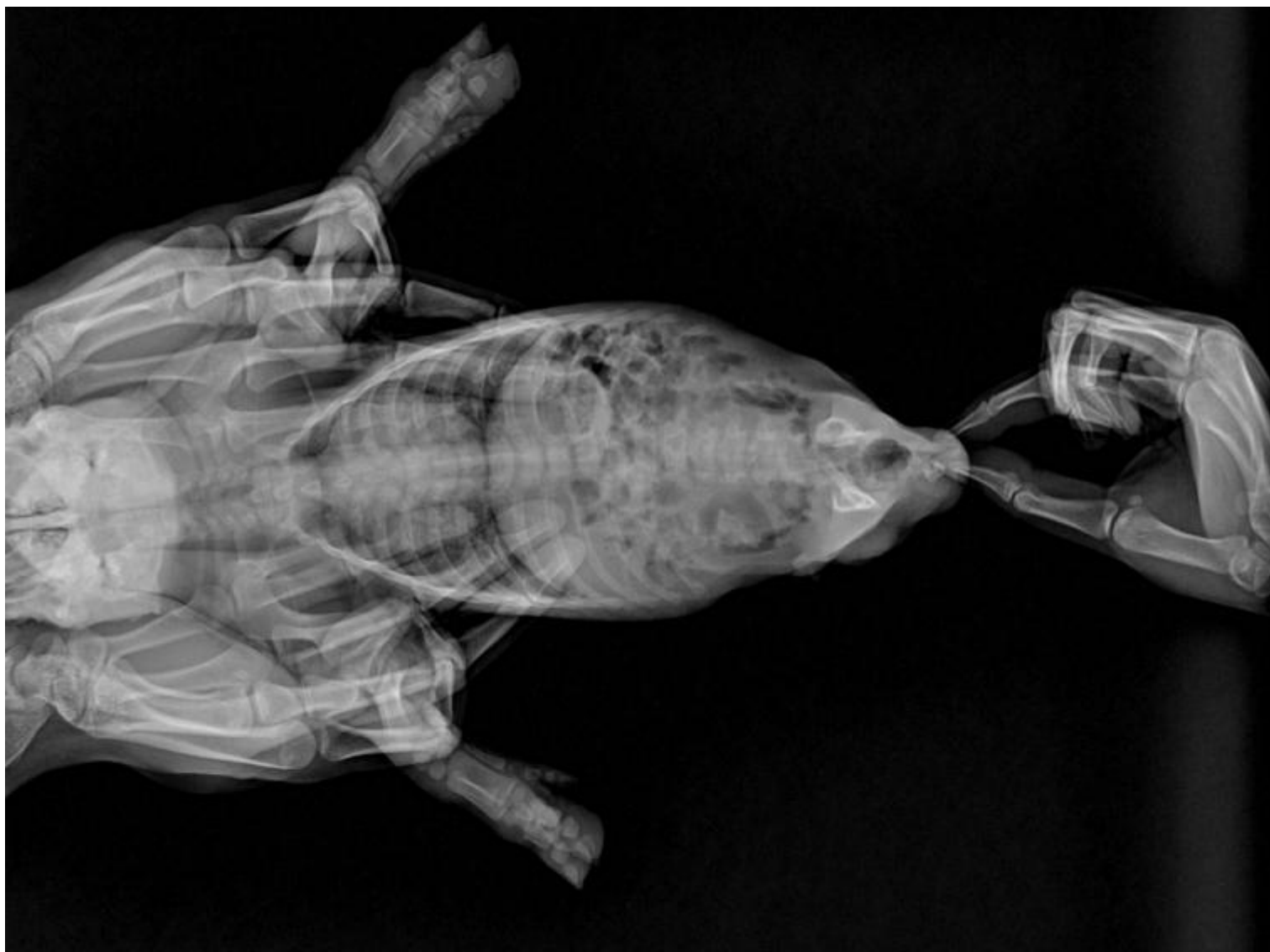

Supplement: Supplementary file 2 — Additional file 1: Fig. S1. X-ray (side view and vertical view) of the handicapped pig (14 days, July 13th, 2020). Human hands were inevitably included in this graph. [file 12864_2021_7925_MOESM1_ESM.pdf]
